# Decision making about Pap test use among Korean immigrant women: A qualitative study

**DOI:** 10.1111/hex.12507

**Published:** 2016-09-30

**Authors:** Kyounghae Kim, Soohyun Kim, Joseph J. Gallo, Marie T. Nolan, Hae‐Ra Han

**Affiliations:** ^1^ School of Nursing University of Connecticut Storrs CT USA; ^2^ Department of Community‐Public Health School of Nursing Johns Hopkins University Baltimore MD USA; ^3^ Department of Mental Health Bloomberg School of Public Health Johns Hopkins University Baltimore Baltimore MD USA; ^4^ Department of Acute and Chronic Care School of Nursing Johns Hopkins University Baltimore MD USA

**Keywords:** cervical cancer screening, decision‐making, immigrant women, qualitative study

## Abstract

**Background:**

Understanding how individuals make decisions about Pap tests concerning their personal values helps health‐care providers offer tailored approaches to guide patients’ decision making. Yet research has largely ignored decision making about Pap tests among immigrant women who experience increased risk of cervical cancer.

**Objective:**

To explore decision making about Pap tests among Korean immigrant women.

**Methods:**

We conducted a qualitative descriptive study using 32 semi‐structured, in‐depth interviews with Korean immigrant women residing in a north‐eastern metropolitan area. Data were audio‐recorded, transcribed verbatim and analysed using inductive coding.

**Results:**

Although most women with positive decisions made their own decisions, some women deferred to their providers, and others made decisions in collaboration with their providers and significant others. While women making positive decisions tended to consider both barriers to and facilitators of having Pap tests, women making negative decisions predominantly discussed the barriers to having Pap tests, such as modesty and differences between the South Korean and US health‐care systems. The women's reflections on their decisions differed regarding their Pap test decisions.

**Conclusions:**

Women's desired roles in the decision‐making process and reflection on their decision outcome appeared to vary, although most participants with positive decisions made their own decisions and were satisfied with their decisions. Future research should conduct longitudinal, quantitative studies to test our findings regarding decision‐making processes and outcomes about Pap tests.

**Implications:**

The findings should be incorporated into cervical cancer screening practices to fulfil the unmet needs of immigrant women in patient‐provider communication and to facilitate women's decision making about Pap tests.

## 1 Introduction

Over the last few decades, there has been considerable discussion about patient engagement in decision making.^1^ The degree to which a patient participates in decision making ranges from active participation in which the patient is empowered to make his/her own decisions about his/her health to passive participation in which the physician decides what is best.^2^ There is now more emphasis on shared decision making as a model of patient‐physician collaboration in health behaviours and outcomes in which more than one possible option exist, such as prostate cancer screening.^1^, ^3^, ^4^

Studies of shared decision‐making in breast, colorectal and prostate cancer screening revealed that patient/provider communications are often ineffective.^5^, ^6^, ^7^, ^8^ Elston Lafata et al.^5^ found that health‐care providers generally acknowledged the importance of shared decision making in cancer prevention and endorsed the importance of discussing risks (64%) and benefits (79%) of tests, yet only about half of respondents endorsed eliciting patients’ preferences. Hoffman et al.^6^ also found that providers did not inquire about patients’ screening preferences and tended not to invite patients to engage in decision making. Patients who discussed cancer screening with their health‐care providers frequently felt uninformed about the screening including drawbacks of testing.^6^ Gaps in communications between providers and patients could negotiate genuine sharedness in the decision‐making process. Hoffman et al.^7^ found that while 68%‐85% of providers expressed opinions about the screening (recommendation), many participants (45%‐69%) considered patients the final decision makers. In some cases, patients made decisions with the aid of their provider (27%‐38%); only a few patients relied on health‐care providers’ opinion alone. Yet, these studies showed that health‐care providers usually failed to encourage a balanced discussion of screening and patients’ preferences.^7^, ^8^ This failing might have reduced the quality of the cancer screening decision and may have precluded patients from making an autonomous decision.

Despite progress in US cancer control through regular cervical cancer screening (ie a Pap test, also called a Pap smear), Korean immigrant women (KIW) suffer from considerable disparities in cervical cancer. They have the second highest cervical cancer incidence rate.^9^, ^10^, ^11^ Only 63% to 68% of KIW had a triennial Pap test (vs 89% of non‐Hispanic whites).^11^, ^12^, ^13^ Studies have examined the correlates of KIW's Pap tests, including English proficiency, physician's recommendations and lower perceived barriers to Pap tests.^14^, ^15^, ^16^, ^17^ However, there has been little discussion of decision‐making to undergo Pap tests. Compared to screenings for prostate cancer for which a screening recommendation is controversial, the benefits of regular Pap tests are well‐known. Nevertheless, updates on screening methods and intervals for cervical cancer over the last few decades may create confusion; the present guidelines recommend that average‐risk women aged 21‐65 years receive a Pap test every 3‐5 years in the USA.^18^, ^19^ Understanding how KIW make a decision about Pap tests in relation to their cultural and personal values can help health‐care providers offer a tailored approach to facilitate their decision making. No known study, however, has explored what contributes to their decision to undergo or not undergo a Pap test, KIW's role in the decision‐making process or how these women reflect on their decision outcome. Our qualitative study was designed to explore KIW's decision making about whether or not to have a Pap test.

## 2 Methods

### 2.1 Study design

We conducted a qualitative descriptive study using 32 semi‐structured, in‐depth individual interviews with KIW residing in the Baltimore‐Washington metropolitan area to explore their decision making about a Pap test. The individual interview facilitates in‐depth exploration of a respondent's perspectives on health behaviours such as cervical cancer screening to construct meaning and is especially useful when the topic is sensitive.^20^, ^21^

#### 2.1.1 Setting and sample

**Figure 1:**
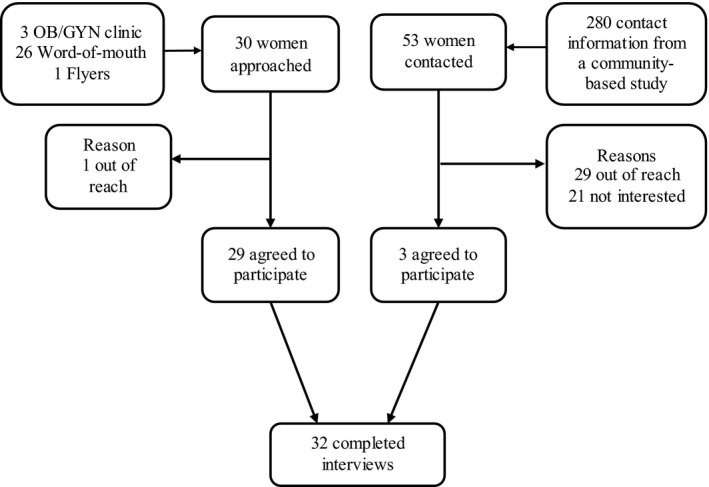
Sampling process

The inclusion criteria were as follows: (i) KIW 21‐65 years of age, (ii) able to read and write in English or Korean; and (iii) had not undergone a hysterectomy. This age range was determined based on the US national cervical cancer screening guidelines.^18^ Participants were recruited from a pool of KIW in the control group from a community‐based randomized controlled trial to promote breast and cervical cancer screenings;^22^ faith‐based organizations; an outpatient obstetrics and gynaecology (OB/GYN) clinic; and by word‐of‐mouth in the Baltimore‐Washington metropolitan area. Recruiting the sample for interviews ended when informational redundancy was achieved.^23^ Figure 1 describes the recruitment process. Using a standardized phone script to achieve a heterogeneous sample, the principal investigator called 53 control group participants in the community‐based trial (n=280), who were selected based on their age, educational level, years of residency in the USA, health insurance status and physician's recommendations.^24^ The principle investigator received 30 unique contacts through distributing flyers in faith‐based organizations and an outpatient OB/GYN clinic, and by word‐of‐mouth. Of 83 potential participants, 21 expressed their lack of interest in the study as a reason for refusal, and 30 were unreachable.

### 2.2 Procedures

The interview guide was prepared with emphasis on KIW's experience with and culture‐specific perceptions of having a Pap test, decision making about Pap tests including their roles in decision making, and confidence and satisfaction with the decisions (see Table 1). In collaboration with community members whose characteristics were similar to study participants, two nurses including the principal investigator developed and finalized the interview guide. The interview guide was prepared in English and was then translated into Korean. The team reviewed all changes.

**Table 1:** Main topics and sample questions used in qualitative interviews

Topic	Sample questions
Experiences with cervical cancer screening	Tell me what comes to your mind when you hear cervical cancer screening testsTell me about your experiences in getting cervical cancer screening tests
Decision‐making process about cervical cancer screening	Could you tell me things that you thought about when deciding about cervical cancer screening?What is the most important reason for your decision about cervical cancer screening?How would you describe your role in the decision‐making in relation to cervical cancer screening?
Reflection on the decision	How confident are you that you made the right decision to have the Pap test?Tell me how satisfied you feel about that decision.Tell me about your future plan for cervical cancer screening.

The study team obtained approval from the institutional review board. After ensuring an understanding of the purpose and potential risks and benefits of the study as well as the voluntary participation of participants, the interviewer obtained informed consent. All interviews were conducted in Korean by a PhD candidate in nursing who was a native Korean speaker and fluent in English. The interviews were conducted in a private place selected for the participants’ convenience, such as their home and a room at a public library. Each interview lasted on average 1 hour (range 30 minutes‐2 hours), and each woman received $30 as a token of appreciation. If needed, a telephone follow‐up was requested and completed to clarify any necessary data. During the interview process, input from participants was added following original discussion by comparing results against prior interviews, which informed the modification of questions in the guide. The confidentiality of participants was maintained throughout the process. The interviewer ensured that all participants were aware of Pap tests prior to the interview. During the interviews, the interviewer took field notes. The interviews were audio‐recorded and transcribed verbatim.

### 2.3 Interview participants

Participant characteristics (N=32) are summarized in Table 2. Approximately 72% of the participants were married and middle‐aged (mean[SD]=48.7[11.8] years). More than half of the participants had some college education and more than 10 years of residency in the USA (mean[SD]=14.4[2.7]), yet had difficulty with English (66%). Nine participants lacked health insurance, and 22% reported receiving a physician's recommendation to obtain Pap tests. Fifteen had received a triennial Pap test, and six had never received one.

**Table 2:** Sample characteristics of qualitative interviews (N=32)

Variables	n (%)	Mean ± SD
Age, years (range=22‐63)		
20‐30s	8 (25.0)	48.7 ± 11.8
40s+	24 (75.0)	
Marital status		
Married/partnered	23 (71.9)	
Separated/widowed/divorced/never married	9 (28.1)	
Years of education (range=9‐20)		
High school graduate or less	14 (43.8)	14.4 ± 2.7
Some college+	18 (56.3)	
Years in the United States (range=0.4‐38.2)		
<25% of their life	13 (40.6)	16.4 ± 9.9
25%+ of their life	19 (59.4)	
Income level		
Very comfortable/comfortable	13 (40.6)	
Neutral/uncomfortable/very uncomfortable	19 (59.4)	
Have health insurance	23 (71.9)	
English proficiency (range=1‐4)		
Not at all/poor/fair	21 (65.6)	2.9 ± 1.1
Fluent	11 (34.4)	
Receive a doctor's recommendation	7 (21.9)	
Latest Pap test use		
≤3 y	15 (46.9)	
>3 y	11 (34.4)	
Never had a Pap test	6 (18.8)	

SD, Standard deviation.

### 2.4 Data analysis

The interviews were analysed using QSR International's NVivo 10 qualitative data analysis software. The analysis drew on borrowed components from the grounded theory methodology:^20^ (i) simultaneous involvement in data collection and reflection on interviews, (ii) open, inductive coding and (iii) memo writing, rather than developing a theory using theoretical sampling and using a constant comparative method. Two bilingual coders read the interview transcripts and field notes several times to develop a general understanding of the interviews and highlight possible categories to explore; the two coders performed the coding process independently. Open, inductive coding was conducted to develop a comprehensive codebook using the first three interviews. Coder agreement rates for each code ranged from 89% to 100%. Discrepancies between the two coders were resolved during team discussions, which is a strategy used to reach a deeper understanding of the data and incorporate diverse perspectives by facilitating discussions between the two coders. Subsequently, the codebook was applied to three interviews to determine whether it fit the data. The codebook was revised based on the identified discrepancies with the data. All transcripts were coded using the finalized codebook and field notes as well as memos, although newly emergent concepts were added to the finalized codebook and applied to the data. The team discussed emerging categories during regular meetings. Memos kept the coders involved in the analysis of the data and helped raise the level of abstraction of the primary coder.

### 2.5 Methodological rigor

To mitigate methodological concerns related to repeated translation, the final results (ie categories, subcategories, relevant quotes) were translated from Korean to English by a professional translator. During the translation process, bilingual researchers on the team met regularly to help incorporate the KIW's emic perspectives in relation to their decision making about Pap tests. Trustworthiness was enhanced^25^, ^26^: (i) the team's prolonged experiences and engagement with KIW promoted credibility, (ii) the thick description of the results such as verbatim transcriptions and subsequent quotes maximized transferability, and (iii) independent coding by two bilingual researchers and the reconciliation of discrepancies among the research team enhanced confirmability.

## 3 Results

Three main categories were as follows: women's preferred role in the decision‐making process; weighing barriers to and facilitators of a positive decision (vs negative decisions, eg decisions not to have Pap tests); and women's reflections on their decision‐making outcome. Each category included several subcategories explaining the process of women's decision making.

### 3.1 Women's preferred role: I would participate in making the decision but may defer to a doctor or collaborate with others

Upon being asked about their role in making a Pap test decision, many respondents paused to think, saying that they had never thought about this before. After the women were probed using the interview guide questions, they were able to explain how they participated in making a Pap test decision. KIW's preferred roles in decision making were autonomous, hierarchical, collaborative (some participants with the doctor and others with their spouse) and peer‐influenced (Table 3).

**Table 3:** Korean American women's desired role in decision‐making process about a Pap test

	Category	Sample quotes
Positive decision	Autonomous	[*Interviewer: Who made the decision?*] Of course I did it. … Yeah, I was in the center of it all. Of course, I got information from my friends and also the news media. When doctors gave me recommendations, I did not just follow them blindly. I did the research first. Through the research, I learned better about the test, understood why it is necessary, and convinced myself to take the examination. So I make my own decisions [Screener].
	Hierarchical	[*Interviewer: When did you make the decision to receive a Pap test?*] I made the decision right after the doctor recommended it. I thought I must take the test because I was sick at the time [Screener].
		After I got diagnosed at the hospital, the doctor said, ‘I recommend you get a Pap test’. Then I took the test [Under screener].
	Collaborative husband	I discuss about the test with my husband. I tell him, ‘I think I may need to get a Pap test’. About once a year, my husband also casually asks, ‘Isn't it about time [to go]?’ We then talk [about the test] [Screener].
	Physician	I don't think I made the decision on my own. The doctor helped me. He suggested first and after talking to him, I thought I should get tested while I am at the hospital. So I think I made the decision with the doctor [Under screener].
	Peer‐influenced	My friend had uterine fibroids and said she had postmenopausal bleeding. So, we were like ‘let's do this together because I had a fibroid too’. That was why we decided to go to the hospital together and got the tests including a Pap test. (Interviewer: So you went to the hospital after talking about it with your friend?) Yes, we made an appointment together and went to the hospital on the same day [Under screener].
Negative decision	Autonomous	The decision not to get a Pap test was made by me. I might have thought about getting one if anyone around me suffered from cervical cancer [Never screener].

#### 3.1.1 Autonomous type

Many participants claimed that, after searching for information from doctors, friends and the media, they made their own decision to have or not have a Pap test. One woman who had never had a Pap test reported, ‘I am the one who made the decision. I made my own decision by myself without asking for help from others [Never screener]’.

#### 3.1.2 Hierarchical type

Some respondents who had a regular resource and hence had received provider's recommendations indicated that they followed his or her advice. Interestingly, this pattern was obvious for women who came to the clinic for noticeable health issues, such as bleeding between periods.

#### 3.1.3 Collaborative type

Respondents made the Pap test decision in collaboration with their health‐care providers or significant others after reviewing the information. Disclosing information on cervical cancer and Pap test use to her husband and inviting her husband to assist in the decision about a Pap test was salient to younger participants. As one young woman stated, ‘I discuss about the test with my husband. I tell him, “I think I may need to get a Pap test.” About once a year, my husband also casually asks, “Isn't it about time [to go]?” We then talk [about the test] [Screener]’.

#### 3.1.4 Peer‐influenced type

Only a few respondents stated that their decision was based on peer influence. However, conversations about cervical cancer and the Pap test occurred only between very close friends. One woman mentioned,

My friend had uterine fibroids and said she had postmenopausal bleeding. So, we were like ‘let's do this together because I had a fibroid too’. That was why we decided to go to the hospital together and got the tests including a Pap test [Under screener].

### 3.2 Weighing barriers to and facilitators of a positive decision to have a Pap test

While participants who had received regular Pap tests (screeners) tended to address both barriers to and facilitators of a positive decision, women who have not received a triennial Pap test (under screeners or never‐screeners) predominantly mentioned the barriers to having the Pap test.

#### 3.2.1 Barriers to a positive decision to have a Pap test

The recurrent barriers were individual and systemic (Table 4). Individual barriers included a lack of awareness/limited knowledge, perceptions and beliefs about Pap test and cervical cancer (eg patient modesty concerns, uterus is expendable, low susceptibility and cancer fatalism), and repeated normal results. Systemic barriers were difficulty finding culturally appropriate providers and a discrepancy between the Korean and the US health‐care systems.

**Table 4:** Barriers to a positive decision to have a Pap test

Category	Sample quotes
Lack of awareness/limited knowledge about cervical cancer and Pap test	The doctor did a test on my uterus and then checked if I had breast cancer, using a machine. That was it. [*Interviewer: Usually they collect the cell using a Q‐tip or a brush on the cervix. And then they put it in a jar and…*] Yes, yes, that's it. So I did get a Pap test [Screener].
Perceptions and beliefs about gynaecological examinations, uterus, cervical cancer	*Modesty*:Even though it's in front of a doctor, I feel extremely humiliated when I spread my legs – I have never done that even in front of my husband. I'm okay with showing other parts like my breasts but I hate to expose myself down there [Under screener].
	*Uterus is Expendable*:I am done having children. So, I didn't worry too much about my uterus. I thought I could just remove it if there is a problem. So I wasn't so concerned [Never screener].
	*Low Perceived Susceptibility*:I'm pretty healthy. I delivered all my children just fine and had menstruation without any pain. So I don't worry so much about OB/GYN checkups [Screener].I am confident that my uterus is healthy and clean [Never screener].
	*Cancer Fatalism*:When she [her sister who has been diagnosed with breast cancer] showed up wearing a wig, I thought my heart stopped pounding. That's how I feel now… I am so sad and just realized, ‘the screening and treatment… are all meaningless. That is cancer [Under screener].
Repeating normal results	Everything was normal when I took the tests. I didn't see why I should get a Pap test again when there wasn't anything positive [Screener].
Difficulties in finding culturally appropriate providers	If possible, I would like my doctor to be competent. It would be nice if she were a woman and even better if she could speak Korean. But it's very difficult to find a doctor who meets all the three conditions, especially at the OB/GYN clinic [Screener].
	Because I am a woman, I would like to have a female doctor if I need one [Never screener].
Discrepancies between the South Korean and US health‐care systems	When I go to the OB/GYN, they tell me to go somewhere else to receive a mammogram… Because the medical system here is all so fragmented, there are many inconvenient situations [such as having to make multiple visits to get a cancer screening done] [Under screener].

OB/GYN, obstetrics and gynaecology.

##### Lack of awareness/limited knowledge about cervical cancer and Pap tests

Many participants did not know what the Pap test is. In fact, some were introduced to the Pap test during the interviews. Not all of the women who received a regular Pap test knew that they had had one, or had a clear understanding of the details (who it is recommended for, how frequently one should have one and pathology associated with cervical cancer). One woman mentioned,

The doctor did a test on my uterus and then checked if I had breast cancer, using a machine. That was it. [*Interviewer: Usually they collect the cell using a Q‐tip or a brush on the cervix. And then they put it in a jar and…*] Yes, yes, that's it. So I did get a Pap test [Screener].

Moreover, most participants noted that their physician did not discuss their test results with them. For example, some KIW stated that they wanted to have more dialogue with doctors about their Pap test results rather than receiving a letter, phone message or no message at all.

##### Perceptions and beliefs about gynaecological examinations, uterus and cervical cancer

Participants shared their perceptions and beliefs about Pap tests and cervical cancer which influenced their decisions about a Pap test. Patient modesty concerns acted as a significant barrier to their agreeing to have a Pap test and made them less likely to obtain a Pap test. One woman stated,

Even though it's in front of a doctor, I feel extremely humiliated when I spread my legs – I have never done that even in front of my husband. I'm okay with showing other parts like my breasts but I hate to expose myself down there [Under screener].

A belief that the uterus is expendable was mentioned by several older women (with children) as a reason against having a Pap test. Most women expressed a belief that they were not a risk for cervical cancer because they had no visible or noticeable symptoms. Some women also mentioned cancer fatalism as a barrier to having a positive decision. Those who had relatives or close friends who had been diagnosed with cervical cancer believed that cancer is not something that could be prevented by screening.

##### Repeating normal results

Participants who have received several Pap tests noted that they had considered not getting a Pap test because of normal Pap test results in the past. As one woman mentioned, ‘Everything was normal when I took the tests. I didn't see why I should get a Pap test again when there wasn't anything positive [Screener]’.

##### Difficulties in finding culturally appropriate providers

While respondents wanted a renowned, Korean‐speaking, female doctor near their home who accepted their health insurance, the majority had not been able to find a culturally appropriate health‐care provider in their community, and therefore had not had a Pap test. However, most women expressed a preference for a non‐Korean‐speaking male doctor when there was no non‐Korean‐speaking female provider in the community. They were afraid of encountering Korean male providers in the Korean community or having friends or relatives who personally knew the male providers.

##### Discrepancies between the South Korean and us health‐care systems

All women were immigrants from South Korea where universal health‐care coverage is offered. One of the biggest challenges at the system level that discouraged these women from getting a Pap test had to do with the fragmented health‐care system in the USA. One woman mentioned,

When I go to the OB/GYN, they tell me to go somewhere else to receive a mammogram… Because the medical system here is all so fragmented, there are many inconvenient situations [such as having to make multiple visits to get a cancer screening done] [Under screener].

#### 3.2.2 Facilitators of a positive decision to have a Pap test

The recurrent facilitators were perceptions about cervical cancer and the Pap test (a belief that cervical cancer will be cured if detected early, fear about cervical cancer, mother should be healthy) and peer pressure (Table 5).

**Table 5:** Facilitators of a positive decision to have a Pap test

Category	Sample quotes
Perceptions about cervical cancer and being a mother	*A Belief that Cervical Cancer Will Be Cured If Detected Early*:Many people say if you get diagnosed at an earlier stage, cervical cancer can be cured and you can get better faster. So, I get myself tested to examine my health [Screener].
	*Fear about Cervical Cancer*:Cancer is terrifying. That's why I should go [Screener].
	*Mother Should Be Healthy*:I need to think of my children… I need to take care of myself to live happily ever after with my children [Screener].I'm a wife and a mother and I believe the happiness of my family depends on my health. So I feel the responsibility to stay healthy [Under screener].
Peer pressure to get a Pap test	I may take courage to have a Pap test if a group of women… like 15 women goes to clinics at the same time [Never screener].

OB/GYN, obstetrics and gynaecology.

##### Perceptions about cervical cancer and being a mother

Participants who had received regular Pap tests believed that cervical cancer could be cured if it was detected early enough. While some women might delay getting a Pap test due to their fear of being diagnosed with cervical cancer, most women who had received a Pap test stated that their fear associated with cervical cancer was a factor in their decision to get a Pap test. In Korean culture, the mother is responsible for taking caring of the entire family, including the children. Therefore, a majority of women in this study thought that they should stay healthy in order to care for their families. One mother stated, ‘I'm a wife and a mother and I believe the happiness of my family depends on my health. So I feel the responsibility to stay healthy [Under screener]’.

##### Peer pressure to get a Pap test

Some women stated that their friends who had received a Pap test regularly inspired them to get a Pap test. One woman stated, ‘My friends take care of themselves a lot better than I do. They go to the hospital religiously, take all kinds of regular checkups and are very committed to their health. I think I should be like them [Screener]’.

### 3.3 Reflecting on the decision outcome

The women's reflections on the decision outcome differed by their Pap test decisions and included the following subcategories (Table 6): being glad to have it done (satisfied); becoming neutral; or being ambivalent for those who decided to have it; or being comfortable with the decision (still confident); or just living with the decision for those who decided against it (indifferent).

**Table 6:** Korean American women's reflection on the decision outcome

Category	Sample quotes
A positive decision about a Pap test	
I Am Glad I Did	I was proud that I was able to go through the health care system to get the examination [in English]. I was glad that I got the test and felt very relieved. I would like to get tested once more [Screener].
Being neutral	Every time I had an examination, the results turned out to be normal. Now I don't feel as nervous as I did before. I just think I will be alright. I still get myself checked regularly but I no longer worry that I might have a problem [Screener].
Being ambivalent	I was like ‘OK, just get over it [Pap test] this time’. But if I were able to go back in time when I made the decision, I might not have gotten the test because I needed to show my private parts to the physician. … But, I became to know the importance of a regular screening after the visit. … Well… I have mixed feelings about the decision [Screener].
A negative decision about a Pap test	
Being confident with the decision	I had done checkups once every few years before I came to America [13 y ago]. Also, I often see my friends going to the OB/GYN because they have an infection in their uterus, but I had never experienced noticeable symptoms. So I am kind of confident about my decision [to not get a Pap test] [Under screener].
Just living with the decision (being indifferent)	I'd like to go [to get a screening], but there's nothing I can do about it. My situation [that I can't take time off from work] is not quite favorable. So I just live with my decision [to not get a Pap test] believing that I will be alright [Never screener].

OB/GYN, obstetrics and gynaecology.

#### 3.3.1 A positive decision about a Pap test: satisfied, neutral or ambivalent

##### I am glad i did and would make the same decision

Most participants who underwent screening were glad that they got a Pap test and reported being satisfied with their decision. Positive post‐decision satisfaction helped the women make the same decision again. One woman who had a Pap test at the free community health clinic stated, ‘I am glad that I was able to go through the health care system to get the examination [in English]. I was glad that I got the test and felt very relieved. I would like to get tested once more [Screener]’.

##### Becoming neutral

Some participants were initially worried and became anxious about their decision when getting a Pap test, but after several Pap tests they became neutral. The effect of ‘being neutral’ on their future decisions appeared to vary. One woman who was aware of importance of a Pap test noted that her decision would not be changed, saying: ‘Every time I had an examination, the results turned out to be normal. Now I don't feel as nervous as I did before. I just think I will be alright. I still get myself checked regularly, but I no longer worry that I might have a problem [Screener]’.

##### Being ambivalent

In contrast, others were ambivalent about their decision due to continuing mental distress related to Pap tests (eg modesty concerns). This mostly occurred among women whose Pap test results were clear.

#### 3.3.2 A negative decision about a Pap test: still confident or indifferent

##### Being confident with the decision

A few women mentioned that they were confident with their decision not to get a Pap test because they did not have any noticeable symptoms, which made them think they had a low risk of developing cervical cancer. One woman mentioned,

I had done checkups once every few years before I came to America [13 years ago]. Also, I often see my friends going to the OB/GYN because they have an infection in their uterus, but I had never experienced noticeable symptoms. So I am kind of confident about my decision [to not get a Pap test] [Under screener].

##### Just living with the decision (being indifferent)

Women who wished to receive a Pap test eventually decided not to get a Pap test because their life situations did not allow them to do so. They said they did not like it, but were willing to live with their decision.

## 4 Discussion

This qualitative investigation offers in‐depth, cultural insights on KIW's decision making about a Pap test. A variety of barriers to and facilitators of a Pap test affected KIW's decisions to take a Pap test. Yet, the degree to which barriers to and facilitators of a positive decision regarding a Pap test influences whether to receive a Pap test or not could be better understood by relating our findings to established decision‐making frameworks. For example, the compensatory model for predicting decision‐making posits that consumers come to a decision by weighing all attributes of a service whereas, in the non‐compensatory model, consumers arrive at a decision by considering certain preferential attributes.^27^, ^28^ In our study, several attributes such as patient modesty concerns, no noticeable symptoms and the absence of culturally appropriate providers in the community have been identified as most pertinent to under‐ and/or never‐screeners’ decisions regarding a Pap test. By the same token, women who were up‐to‐date on their triennial Pap test appeared to consider multifaceted factors in relation to Pap tests. Yet, no known study has attempted to predict decision processes among KIW based on decision‐making frameworks. Our emergent hypotheses should be tested using quantitative research to examine whether non‐compensatory models (eg lexicographic and elimination‐by‐aspects strategies) explain decisions about a Pap test among under‐ and never‐screeners and whether compensatory models explain decisions regarding a Pap test among women with up‐to‐date screening. Future research should also examine what factors predict the choice of type of Pap test decision‐making models such as the levels of health literacy.

Charles and her colleagues^3^ identified three prototypes of shared decision making in medical encounters: (i) a paternalistic model in which the decision is made by providers, (ii) an informed model in which the decision is made by patients after reviewing alternative options, and (iii) a shared decision‐making model in which the decision is made collaboratively by providers and patients on the basis of shared information. We found that all three decision patterns were noted, which is compatible with our previous study on the human papillomavirus vaccination among KIW.^26^ However, most women in this study appeared to be drawn to an informed model (autonomous type), although there was a range of breadth and depth of seeking information and sharing the information with another significant decision maker. Dieng et al.^29^ reported that most Australian women preferred to be actively involved in decision making about routine Pap tests (87%) and that they would require information about the benefits and harms of a Pap test prior to the screening. In contrast, Greek patients with breast cancer preferred a passive role in cancer treatment decision making.^1^ It is noteworthy that healthy women appear to be favourable to taking control of their health by actively engaging in the decision making when options and consequence of their decision are clear. Some KIW reported making decisions based on a paternalistic model (hierarchical type). This result might be associated with the fact that most of the women did not have regular health‐care sources and, hence, tended to make decisions about the Pap test prior to patient‐provider encounters, which results in a hybrid decision type such as a peer‐influenced type in which KIW's decisions were influenced by peers in the community. Indeed, in collectivist cultures such as those of Korea and China, people tend to be interdependent, and they base their behaviour or decisions on community norms.^30^, ^31^ In some cases, collectivism has an impact on emphasizing collective coping that helps individuals overcome unpleasant events^33^ such as getting a Pap test. In fact, under‐ or never‐screeners in this study stated they would have a Pap test if a group of KIW went to clinics at the same time. The role of interdependence in enduring an unpleasant but necessary procedure such as Pap tests could offer an unprecedented chance to promote the Pap test among immigrant women like KIW who come from collectivist cultures.

The intersections of patient modesty and challenges locating culturally appropriate health‐care providers in the Korean immigrant community appeared to pose substantial barriers to decisions to have a Pap test. Modesty appeared to play a critical role in avoiding preventive care such as mammogram^32^ and Pap test^33^ when patients do not exhibit significant symptoms. For some, they might decide not to seek medical care at all due to modesty even if they notice concerning symptoms. Nurses who are trained to see patients holistically are ideally positioned to respect patients’ modesty. As a direct health‐care provider and a bridge to procedures that require patients to expose their genitalia, nurses should encourage patients to express their concerns before and during the medical encounters by carefully assessing patients, building rapport and offering comfortable environments. In addition, there is an urgent need to help the women navigate the health‐care system to gain access to culturally appropriate providers such as same‐gender health‐care providers. Ample evidence supports that a community‐based programme including access‐enhancing strategies is successful in promoting cervical cancer screening in women from diverse racial/ethnic groups including KIW.^22^, ^34^, ^35^ While the ways in which these access‐enhancing programmes promote cervical cancer screening may vary, recent research reports the utility of a community health worker as a navigator, in addition to a health literacy‐focused intervention to promote breast and cervical cancer screening among KIW.^22^ Given that navigation services using a community health worker model were successful in promoting cancer screening among KIW, future research needs to address whether these navigation services can help to match KIW's preference for providers with providers’ characteristics.

Our findings on decision making have implications for practices. In addition to known cultural descriptors – low perceived susceptibility, and cancer fatalism^36^, ^37^ – the perception about the uterus being expendable is partially consistent with a previous qualitative exploration on symbolic meanings of the cervix among KIW. Most middle‐aged KIW in that study linked the cervix and uterus to childrearing; younger women linked it to femininity.^37^ Our study participants believed after they had completed their families, the uterus was no longer necessary and this belief hindered them from getting a Pap test. Studies have reported that mothers within the Korean immigrant community play a significant role in decisions on matters such as their children's health.^26^, ^38^ Similarly, in our study, mothers acknowledged their responsibilities for childcare, which affected their views on their own health. The distinctive cultural views on cervix, cervical cancer and Pap tests should be incorporated into practices for cervical cancer screening, which can then facilitate KIW's involvement in decisions about Pap tests.

Another practical implication is associated unmet needs of KIW in relation to communication with their providers regarding cervical cancer screening practices, which facilitates patient autonomy in the decision‐making process. In this study, nearly half of women who underwent screening did not know that they had received a Pap test, and most participants reported they wanted more conversation about their results with their providers rather than receiving a letter or a message. The findings indicate the KIW did not have adequate discussions about a Pap test before or after the procedures. This finding calls for better patient‐provider communication about cervical cancer and Pap tests among KIW. Evidence suggests that enhanced patient‐provider communication increases the likelihood of receiving certain types of cancer screening such as mammogram and Pap test among 605 predominantly white women aged 40‐75 years^39^ and faecal occult blood testing screening in a nationally representative sample of the US adults aged 50 and older (88% white).^40^ Yet, no study has examined the role of patient‐provider communication in Pap test use among women with limited proficiency in English. Future research is warranted to identify intervention strategies to promote patient‐provider communication among limited English proficient Asian immigrants such as KIW in order to enable them to understand what the Pap test is and why (or why not) they should have one.

### 4.1 Limitations

We recruited participants from one ethnic group in a single metropolitan area, which may limit the applicability of the findings beyond the study sample. However, the purpose of this study was to understand the cultural descriptors of decision making in relation to Pap test use among KIW, who have been underrepresented in the cervical cancer screening literature. We also used a thick description strategy by presenting contextual factors and categories with sample quotes to help readers judge transferability beyond the study sample.

Another limitation has to do with potential recollection bias. The participants were asked to reflect on their experiences in relation to their decisions to receive a Pap test which happened from a few months to several years ago. Some participants had difficulty remembering or articulating their decision making about having a Pap test; these women were given the time to think about their decisions and were then probed using questions from the interview guide. This may have led the result to suggest a more rational model for KIW making decisions about Pap tests. However, a retrospective decision‐making approach may have prevented social desirability bias that can be caused by a priori discussion regarding their decision about a Pap test.

## 5 Conclusions

Understanding factors influencing decision making, women's desired role in decision‐making process and reflection on their decision outcome is a first step in developing a patient‐centred decision‐making intervention programme salient to this population, thereby facilitating KIW's desired role in shared decision making. We made recommendations for best practice in controlling cervical cancer among KIW based on the gaps between KIW's unmet needs and current practice in relation to cervical cancer screening. Future research should also consider longitudinal, quantitative studies to examine how decision‐making processes and outcomes influence KIW's decision to undergo (or not) a Pap test.

## Acknowledgement

Dr. Kim was with Johns Hopkins University when the work was conducted.

## Funding Statement

Financial support for this study was provided in part by a grant from the National Cancer Institute (R01CA129060, Clinical Trials Registry NCT00857636) and was supported by a small grant from the Sigma Theta Tau International, a research award from the Sigma Theta Tau International Nu Beta Chapter, and a dissertation grant from the Fahs‐Beck Fund for Research and Experimentation. The funding agreement ensured the authors’ independence in designing the study, interpreting the data, writing and publishing the report.

## Conflict of Interest

The authors declared no conflict of interest.
